# Fabrication of Poly(butylene succinate)/Carbon Black Nanocomposite Foams with Good Electrical Conductivity and High Strength by a Supercritical CO_2_ Foaming Process

**DOI:** 10.3390/polym11111852

**Published:** 2019-11-10

**Authors:** Zhou Chen, Junfeng Hu, Jiajun Ju, Tairong Kuang

**Affiliations:** 1 School of Mechanical and Power Engineering, Nanjing Tech University, Nanjing 211800, China; zchen6240@njtech.edu.cn (Z.C.); junfeng-hu@njtech.edu.cn (J.H.); 2 Suzhou Yi He Yong Li New Energy Co., Ltd, Suzhou 215400, China; 3 The Key Laboratory of Polymer Processing Engineering of Ministry of Education, South China University of Technology, Guangzhou 510640, China; 201710100572@mail.scut.edu.cn; 4 College of Material Science and Engineering, Zhejiang University of Technology, Hangzhou 310014, China

**Keywords:** poly(butylene succinate) (PBS), carbon black (CB), solid-state ScCO_2_ foaming, lightweight, good electrical conductivity

## Abstract

Lightweight, high-strength and electrically conductive poly(butylene succinate) (PBS)/ carbon black (CB) nanocomposite foams with a density of 0.107–0.344 g/cm^3^ were successfully fabricated by a solid-state supercritical CO_2_ (ScCO_2_) foaming process. The morphology, thermal and dynamic mechanical properties, and rheological behavior of the PBS/CB nanocomposites were studied. The results indicate that the CB nanofiller was well dispersed in the PBS matrix and the presence of a proper CB nanofiller can accelerate the rate of crystallization, improve the thermal stability, enhance the stiffness, and increase the complex viscosity of PBS/CB nanocomposites. These improved properties were found to play an important role in the foaming process. The results from foaming experiments showed that the PBS/CB nanocomposite foams had a much smaller cell size, a higher cell density, and a more uniform cell morphology as compared to neat PBS foams. Furthermore, the PBS/CB nanocomposite foams also possessed low density (0.107–0.344 g/cm^3^), good electrical conductivity (~0.45 S/cm at 1.87 vol % CB loading), and improved compressive strength (108% increase), which enables them to be used as lightweight and high-strength functional materials.

## 1. Introduction

To minimize environmental pollution and the depletion of fossil oils, biodegradable polymeric foams are increasingly being considered as a promising alternative to replace the petroleum-based polymeric foams [1,2,3,4,5,6]. Over the last few decades, many biodegradable polymers, such as poly(lactic acid) (PLA) [7], polycaprolactone (PCL) [8,9], polyhydroxyalkanoates (PHAs) [10,11], and poly(butylene succinate) (PBS) [6], have been extensively studied. Compared to PLA, PCL, PHAs, and other biodegradable polymers, PBS is regarded as the most promising biodegradable polymer materials due to its good processability and mechanical properties, better thermal and chemical stability, and lower materials cost [12,13,14,15] Due to these good characteristics, PBS materials were expected to produce high-performance biodegradable polymeric foams for various applications. Unfortunately, the linear molecular chains and low molecular weight of PBS itself resulted in a low melt strength, which eventually led to the poor foamability of PBS foams [16].

It is well known that the melt strength of materials is associated with melt viscosity. Therefore, many attempts have been made to improve the melt strength of PBS materials, such as applying chemical reagents or incorporating nanoparticles [15,17,18,19,20]. Using crosslinking or a branching agent to modify the chemical structure of PBS materials was regarded as an efficient route to increase the PBS’s viscosity in many previous studies. For examples, Zhou et al. modified PBS through reactive melt mixing with a chain extender to improve the molecular weight and polydispersity index (PDI), and the volume expansion ratio of the resulting PBS foams was found to reach up to 14.3 [15]. Pentrakoon et al. found that a lower degree of crystallinity, a higher viscosity, and a more highly crosslinked structure could be achieved in PBS by the addition of dicumyl peroxide (DCP) or N3300, which consequently improved the foamability of PBS [18]. Great achievements have been made using the chemical modification strategy; however, some adverse factors (e.g., reagent toxicity, negative to its biodegradation property, etc.) cannot be ignored. The use of nanofillers, such as multi-walled carbon nanotube (MWCNTs) [21], halloysite nanotubes (HNTs) [19], and carbon nanofibers (CNFs) [22] has been reported to be a successful way to improve the melt viscosity, crystallization behavior, and foamability of PBS materials. However, the cost of the above-mentioned nanofillers is relatively high. Hence, there remains great room to improve PBS’s foamability by adding inexpensive nanofillers.

Carbon black (CB) is a cheap and widely used carbon nanoparticle that possesses good electrical conductivity, a high specific surface area, and a favorable absorption ability [23,24,25]. Therefore, incorporating CB nanofiller into a PBS matrix can be expected to improve the foamability of the PBS and endow the nanocomposites and nanocomposite foams with good electrical conductivity. In fact, electrically conductive polymer composite foams have attracted a great deal of interest over the past few decades. The introduction of microcellular structure would not only reduce a sample’s density but also improve the sample’s electrical conductivity. [26,27,28] Some electrically conductive PBS-based composite foams works have been reported [6,29], but the loadings of conductive filler (e.g., carbon fiber) and the percolation threshold are still high. Therefore, the aim of this work is to prepare lightweight high-strength PBS nanocomposite foams with low addition of cheap conductive nanofiller, good electrical conductivity and low percolation threshold for electronics applications. Firstly, a series of PBS nanocomposites with different contents of inexpensive CB nanofillers were fabricated by a direct melt-compounding approach. Then, their nanocomposite foams were prepared by a solid-state supercritical CO_2_ foaming process. The morphological characteristics, thermal, crystallization and dynamic mechanical properties, and rheological behavior of the PBS/CB nanocomposites were studied. The foaming behavior and mechanical compression properties of various nanocomposite foams were analyzed. Lastly, the electrical conductivity of the PBS/CB nanocomposites and their nanocomposite foams is discussed.

## 2. Experimental Part

### 2.1. Raw Materials

PBS (B601) was supplied by Anqing He Xing Chemical Co., Ltd. (Anqing, China). Carbon black (CB) (30–45 nm) was purchased from Nanjing Xianfeng Nanotech Co. Ltd. (Nanjing, China). Commercial CO_2_ (purity 99.99%) was supplied by Guangzhou Shengtong Trading Co. Ltd. (Guangzhou, China).

### 2.2. Preparation of PBS/CB Nanocomposites

**Figure 1:**
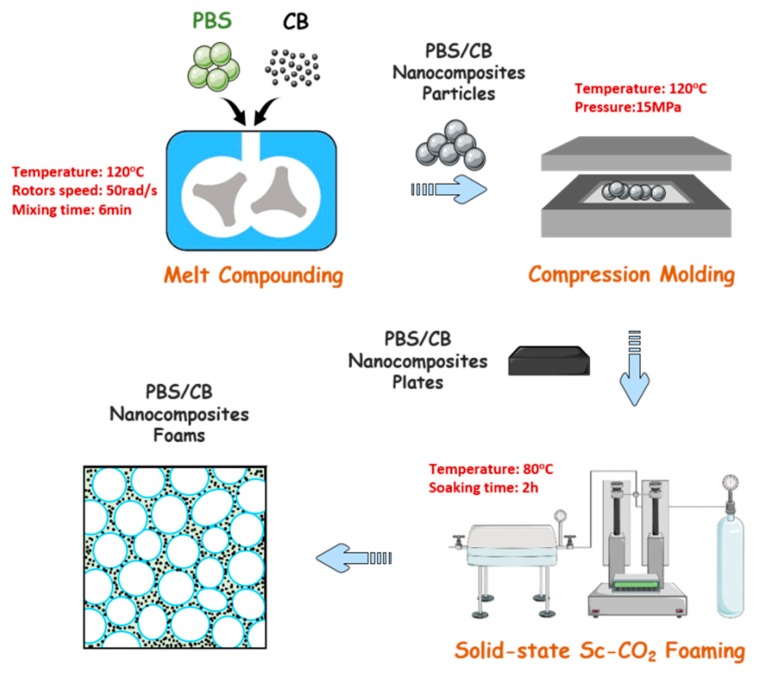
Schematic illustration of the preparation of lightweight microcellular poly(butylene succinate) (PBS)/carbon black (CB) nanocomposite foams.

A schematic illustration of the preparation route is shown in Figure 1. Firstly, PBS pellets and CB nanofiller were both dried at 60 °C for 12 h before melt-compounding. Then, the PBS nanocomposites containing various CB loadings (0.5–10 wt %) were prepared via a melt-mixing method using a laboratory torque mixer (Guangzhou Putong Testing Co., Ltd., Guangzhou, China) with a screw speed of 50 rpm at 120 °C for 6 min. Lastly, the obtained samples were compression molded into a rectangular sheet with a thickness of 1 mm at 120 °C for 5 min under 15 MPa. For convenience, the samples were labeled PBS, PBS0.5, PBS1, PBS3, PBS5, and PBS10 according to the CB concentration.

### 2.3. Preparation of PBS/CB Nanocomposite Foams

The PBS/CB nanocomposite foams were prepared by a solid-state supercritical CO_2_ foaming technique (Figure 1). Firstly, PBS/CB nanocomposites were saturated with Sc–CO_2_ in a laboratory high-pressure autoclave at 80 °C (below the melting point of PBS) and 20 MPa for 2 h to ensure equilibrium adsorption. Subsequently, the autoclave was rapidly depressurized to ambient pressure, and the foamed sample was obtained.

### 2.4. Characterization

#### 2.4.1. Thermal Characterization

Differential scanning calorimetry (DSC) measurements were conducted using a DSC Q20 (TA Instruments, New Castle, DE, USA) under a nitrogen flow. The heating and cooling temperature rate was both set to 10 °C /min. Approximately 3–5 mg specimens of the PBS/CB nanocomposites were heated from 25 to 120 °C, held at 120 °C for 3 min to eliminate the heat history, and then cooled to room temperature. After that, the nanocomposites were reheated to 120 °C again and the DSC test curves were obtained. TGA testing was carried out with a TA Instruments Discovery TGA 550 (New Castle, DE, USA), and each TGA test was conducted at a heating rate of 10 °C/min from 25 to 500 °C under a nitrogen gas atmosphere. A minimum of three samples for each nanocomposite were tested.

#### 2.4.2. Mechanical Property Test

A dynamic mechanical analysis (DMA) test was performed using METTLER DMA 1 in the tensile mode. The PBS/CB nanocomposites were tested from −70 °C to 60 °C at heating rate of 5 °C /min and at a frequency of 1Hz. The specimen dimensions were 30 mm × 10 mm × 1 mm. In addition, the compressive properties of PBS/CB nanocomposite foams were tested using an Instron 5967 (Norwood, MA, USA). Three samples for each foam were tested.

#### 2.4.3. Rheological Test

The rheological properties of PBS/CB nanocomposites were examined with an MCR 302 dynamic oscillatory viscometer (Anton Paar, Graz, Austria) with a parallel plate of 25 mm in diameter. This experiment was performed with a frequency sweep from 0.06 to 628 rad/s at 120 °C with 10% strain level. A minimum of three samples for each nanocomposite were tested.

#### 2.4.4. Electrical Conductivity Test

A high resistance meter (ZC36, Shanghai Anbiao Electronic Co., Ltd., Shanghai, China) and four-point probe (RTS-9, Guangzhou Four-Point Probe Technology Co., Ltd., Guangzhou, China) were used to measure the electrical conductivity of PBS/CB nanocomposites and their nanocomposite foams, respectively. At least five positions were measured on the samples’ surface to obtain the mean value of the electrical conductivity.

#### 2.4.5. Microstructure Analysis

The fracture morphology of PBS/CB nanocomposites and their nanocomposite foams were examined by a field-emission scanning electron microscope (ZEISS Merlin, Germany) operated at an accelerating voltage of 10 kV. The average cell size was obtained by analyzing the SEM images with Image-pro Plus 6.0 software (Media Cybernetics Inc., MD, USA). The cell diameter of at least 50 cells was calculated to acquire the mean value. The average cell density N_0_ (cells/cm^3^) was calculated using the equation below:

(1) $$N_{0}=\frac{6\left[1-\left(\frac{\rho_{f}}{\rho_{s}}\right)\right]}{{\pi D}^{3}}{\times 10}^{12}$$

where *D* (μm) is the average cell size, and *ρ_f_* (g/cm^3^) and *ρ_s_* (g/cm^3^) are the densities of the unfoamed and foamed PBS/CB nanocomposites, respectively. 

## 3. Results and Discussion

### 3.1. Effect of CB Content on the Physical Properties of PBS/CB Nanocomposites

**Figure 2:**
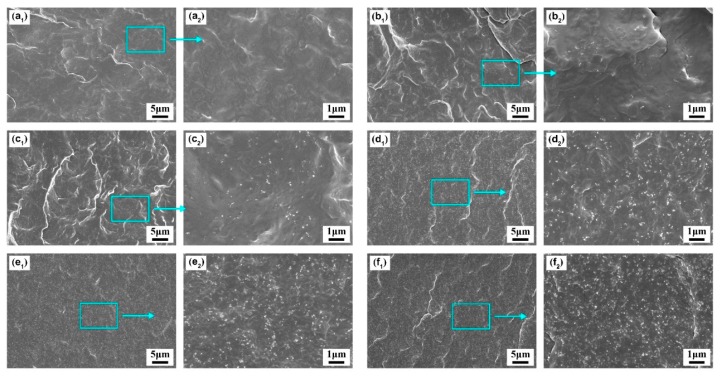
SEM micrographs of PBS/CB nanocomposites: (**a_1_**,**a_2_**) PBS0; (**b_1_**,**b_2_**) PBS0.5; (**c_1_**,**c_2_**) PBS1; (**d_1_**,**d_2_**) PBS3; (**e_1_**,**e_2_**) PBS5; (**f_1_**,**f_2_**) PBS10.

To evaluate the filler’s dispersion in the PBS matrix, SEM was used to observe the fracture surface of PBS/CB nanocomposites. The micrographs are shown in Figure 2. The CB particles’ aggregation size distribution is shown in Figure S1. From these micrographs, it can be seen that the CB fillers were dispersed well in the PBS nanocomposites. As for the neat PBS, some wrinkled structures were found on the surface due to the toughness of the PBS itself. Regarding the PBS/CB nanocomposites, many tiny white dots were observed on surface of the nanocomposite with the addition of CB filler, and such white dots were well embedded in the matrix even at higher CB contents. With the increase in CB content, CB particles gradually agglomerated, and the average size of the agglomeration increased to 204.8 nm. In addition, the fractures became smoother with an increase in the CB loading, which was due to the incorporation of the rigid carbon black filler, which reduced the toughness of the nanocomposites. Nevertheless, the increase in filler concentration was found to decrease the distance between two fillers, which can help the CB nanofiller to form a percolation network in the polymer matrix.

**Figure 3:**
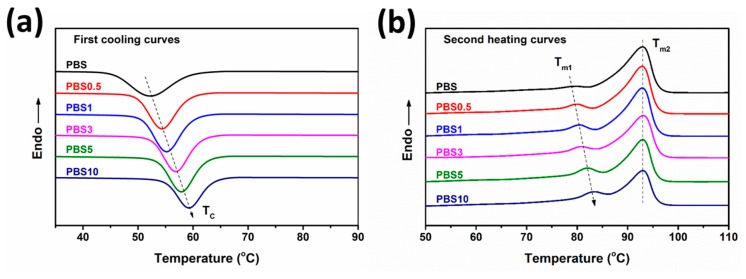
DSC curves of neat PBS and PBS/CB nanocomposites: (**a**) first cooling curve; (**b**) second heating curve.

The thermal and crystallization performance of various PBS/CB nanocomposites were studied by DSC and TGA. Figure 3 presents the DSC curves of neat PBS and PBS/CB nanocomposites. The results of the DSC measurements are presented in Table S1. The crystallization temperature (*T*_C_) of PBS/CB nanocomposites was increased with increasing CB content, from 52.1 °C for pure PBS to 59.2 °C for PBS10, indicating that the heterogeneous nucleation effect of CB nanofiller actually improves the nucleation efficiency of the PBS crystallization process. As shown by the second heating curve, a double-melting peak was observed for PBS, which was attributed to the melting—recrystallization or the melting of incomplete crystals (or crystal slices) during the heating process. *T*_m1_ shifted to a higher temperature with increasing CB content, while *T*_m2_ remained the same. The change in *T*_m1_ was attributed to the heterogeneous nucleation effect of CB nanofiller. The addition of CB nanofiller into the PBS matrix promoted crystallization and increased the crystallinity of the PBS, which resulted in a more complete crystal structure and a higher initial melting temperature; *T*_m2_ mainly corresponded to the intrinsic crystal of the PBS. Similar results have been obtained in previous studies [30,31,32]. 

**Figure 4:**
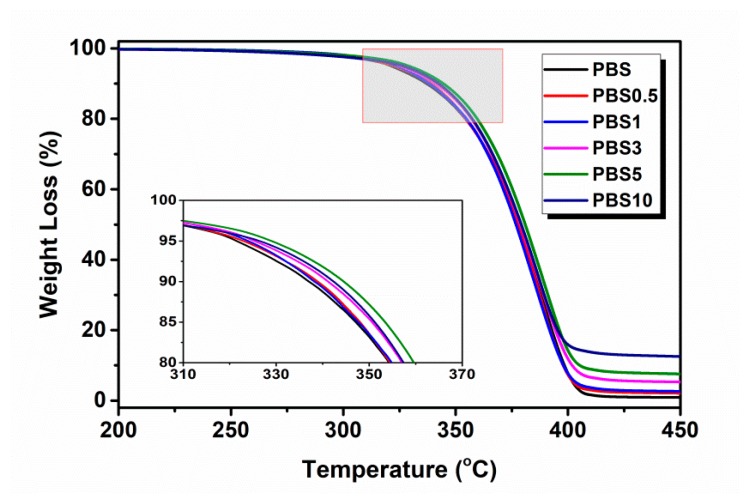
TGA curves of neat PBS and PBS/CB nanocomposites.

Figure 4 shows the TGA curves of the neat PBS and its nanocomposites containing different contents of CB nanofiller. Further details are shown in Table 1. It was observed that the amount of residue of PBS/CB nanocomposites was increased with increasing CB content, which means that such residue originated from the mixture of CB and PBS carbide after thermal decomposition. Compared with the neat PBS curves, all *T*_5%_ values of the PBS/CB nanocomposites were increased with the CB content within the experimental error. During the early stage of thermal destruction, the uniformly dispersed CB served as a heat barrier in the PBS matrix to restrict the movement of PBS molecules and the evaporation of thermal decomposition products [6,31]. By analyzing the *T*_max_ (the maximum decomposition temperature) of PBS/CB nanocomposites, it can be seen that CB promotes the destruction of PBS/CB nanocomposites at high temperatures. CB’s heat barrier effect is more obvious when the added CB content is 5 wt %.

**Figure 5:**
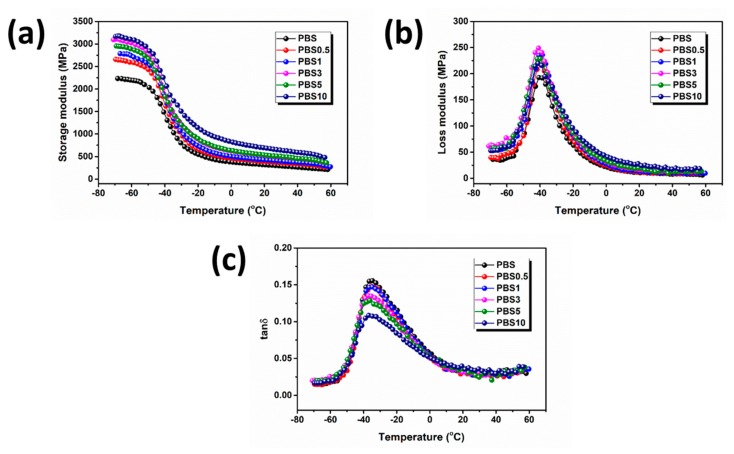
Dynamic mechanical properties of PBS/CB nanocomposites: (**a**) storage modulus (*E*’); (**b**) loss modulus (*E*″); (**c**) tanδ.

Figure 5a shows the DMA curves of storage modulus (*E*’) versus temperature for all the nanocomposites. As expected, all of the PBS/CB nanocomposites had a higher storage modulus than the neat PBS over the whole temperature range, and it increased with increasing CB content. As shown in Figure 5b, the loss modulus (*E*″) values increased initially and then decreased with increasing CB content. This result suggests that the stiffness of the PBS/CB nanocomposites was continuously enhanced by increasing the CB content while their toughness was first increased and then subsequently decreased. This was because CB nanofiller is rigid. At a low loading, both the stiffness and toughness of the PBS/CB nanocomposites improved. When the added CB content was too high, CB nanoparticle aggregation compromised the toughness of the PBS/CB nanocomposites. Figure 5c shows the characteristics of tanδ against temperature. It is clear that adding CB to PBS decreased the tanδ peaks of the PBS/CB nanocomposites, indicating that their damping characteristics had been lowered. This result suggests that, since CB nanofiller has promising damping characteristics, increasing its content decreases the tanδ peak of PBS/CB nanocomposites. Generally, the temperature of a tanδ peak indicates a material’s glass transition temperature. It was observed that the *T*_g_ of the PBS/CB nanocomposites decreased (a minor variation) with an increase in CB concentration. This result indicates that the chain segment of the PBS/CB nanocomposites moved at a lower temperature compared with neat PBS. A similar phenomenon was also observed in a previous study [28]. This is because the free movement of PBS chains was restricted by the rigid dopant CB, resulting in the PBS/CB nanocomposites having an increased storage modulus and a decreased *T*_g_.

**Figure 6:**
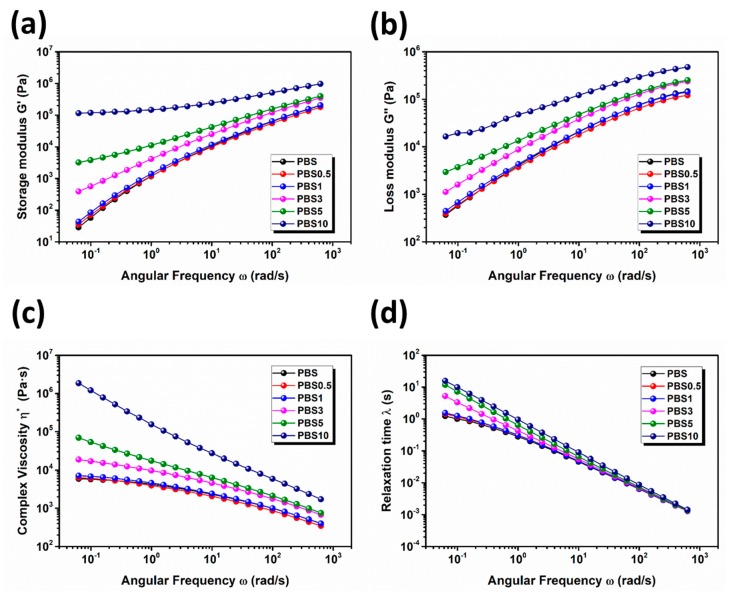
(**a**) Storages modulus (*G*’), (**b**) loss modulus (*G*″), (**c**) complex viscosity (η*), and (**d**) relaxation time (λ) of PBS/CB nanocomposites as a function of angular frequency at 120 °C.

It is well known that the viscosity of the polymer plays an important role in the Sc-CO_2_ foaming process [33,34]. Therefore, the complex viscosity (η*), storage modulus (*G*’) and loss modulus (*G*″) of the PBS/CB nanocomposites were studied. As shown in Figure 6a,b, the neat PBS exhibited the rheological behavior of a homopolymer, and its molecular chains were fully relaxed at a low CB content. When the added CB content was 0.5 and 1 wt %, respectively, no obvious variation tendency was observed for the PBS/CB nanocomposites. However, when the added CB content was increased to 3 wt %, the dependence of the angular frequency on the storage modulus *G*’ tended to decrease. When further increasing the CB content (e.g., 10 wt %), a plateau was clearly observed for the PBS/CB nanocomposites in the low angular frequency region, indicating that the rheological behavior of the nanocomposites had changed from liquid-like behavior to solid-like behavior. Percolation is defined as a phase-transition at which a dramatic change occurs in one parametric value, as this parameter is continuously changed. When the CB content reaches the percolation threshold, the dispersed nanofiller in the polymer matrix tends to form a network structure. We can confirm the successful formation of a CB percolation network in the PBS by the variation in the storage modulus of the PBS/CB nanocomposites. Figure 6c shows the complex viscosity of the PBS/CB nanocomposites as a function of frequency. PBS, PBS0.5, and PBS1 had representative non-Newtonian fluids, showing a narrow plateau in the low frequency region. As the frequency increased, the shear-thinning phenomenon was observed. On the other hand, in the low angular frequency region, the complex viscosity of PBS3, PBS5, and PBS10 continuously increased. This was because the free flow of PBS polymer in the molten state was compromised by the rigid CB nanofiller. This statement will be confirmed in our later discussion about the sample’s foaming behavior. Meanwhile, PBS/CB nanocomposites with high CB contents exhibited shear-thinning across the whole frequency region; this was especially the case for PBS10. The relaxation time of PBS/CB nanocomposites can be calculated by the equation below, and the results are shown in Figure 6d:

(2) $$\lambda =\frac{G^{\prime }\left|\eta^{*}\right|}{{\left(\left|\eta^{*}\right|\omega \right)}^{2}}$$

It was observed that as the frequency increased, the relaxation time of the PBS/CB nanocomposites decreased. This observation can be explained by the fact that the relaxation time will generally be increased when there are a large number of macromolecules or physical structures in the host materials. In the low frequency region, the relaxation time increased as the added CB content increased. This tendency was much more obvious when the added CB content was higher than 3 wt %. This was because the increasing amount of CB made the interaction between CB particles, or the interaction between the CB and the host has a leading effect, which consequently affected the relaxation behavior of the PBS/CB nanocomposites. The solid-like rheological behavior of PBS/CB nanocomposites with high CB contents in a molten state partially contributes to the above fact.

### 3.2. Effect of CB Content on the Cell Morphology and Cell Size Distribution of Nanocomposite Foams

**Figure 7:**
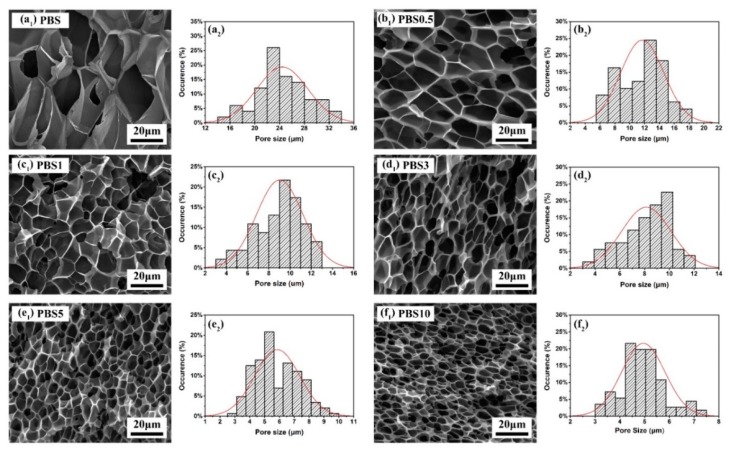
SEM pictures of unfoamed and foamed PBS and PBS5 nanocomposites foams at different intermediate pressure conditions.

**Figure 8:**
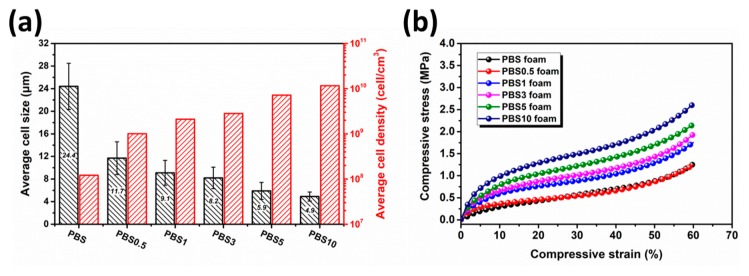
Characterization of neat PBS foams and PBS/CB nanocomposite foams: (**a**) average cell size and cell density; (**b**) compressive strain-stress curves.

Figure 7 displays the cell morphology and size distributions of the PBS/CB nanocomposite foams. The average cell size and average cell density are summarized in Figure 8a. By comparing SEM images and cell size distributions, it was observed that, due to its low viscosity, the neat PBS had a higher expansion ratio and a lower sample density, with an average cell size of 24.4 ± 4.1 μm. As the CB content increased, the average cell size was decreased to 4.9 ± 0.8 μm, and the cell density was increased as well, but the expansion ratio was decreased. The reason for these phenomena was the fact that an increase in the viscosity of PBS/CB nanocomposites will restrict the growth of cells during the Sc–CO_2_ foaming process, thereby affecting the final sample’s density. As mentioned above, CB was distributed in the PBS homogeneously. According to classical nucleation theory and the Gibbs free energy [35,36], the Gibbs free energy at the CB–PBS interface was lower than that in the PBS matrix. Therefore, CB particles acted as the nucleation points of the heterogeneous phase due to the lower activation energy barrier. Their heterogeneous nucleation effect can improve the cell density during the foaming process, which decreases the cell size and improves the uniformity of the cells’ distribution. By observing these SEM images, it was found that the open porosity increased as the CB content increased. This was because cells tend to nucleate heterogeneously. During the cell growth process, the distance between cells is decreased due to the increase in the nucleation density of the cells. If there exists CB nanofiller between the walls of two neighboring cells, these cells may be breached by CB during their growth process, which will make the cell walls thinner. As a consequence, the opening percentage will be increased.

The representative compressive stress-strain curves for PBS/CB nanocomposite foams are shown in Figure 8b. The increasing CB content significantly improved the compressive strength of the nanocomposite foams. For neat PBS foam, the compressive strength was 1.26 MPa. When the CB content increased up to 10 wt %, the compressive strength of the nanocomposite foams showed a 108% increase to 2.63 MPa. The enhanced compressive mechanical performance is ascribed to the following reasons: On the one hand, cell size was decreased and sample density was increased, which makes cell walls stronger to collapse during compression process, resulting in improved compressive strength; on the other hand, the rigid CB nanofiller had a reinforcing effect as well.

The representative compressive stress–strain curves for the PBS/CB nanocomposite foams are shown in Figure 8b. The increase in CB content significantly improved the compressive strength of the nanocomposite foams. For the neat PBS foam, the compressive strength was 1.26 MPa. When the CB content was increased to 10 wt %, the compressive strength of the nanocomposite foams showed a 108% increase to 2.63 MPa. The enhanced compressive mechanical performance can be ascribed to the following reasons: on the one hand, the cell size was decreased and the sample’s density was increased, which makes cell walls less likely to collapse during the compression process, resulting in improved compressive strength; on the other hand, the rigid CB nanofiller also had a reinforcing effect.

### 3.3. Electrical Conductivity of PBS/CB Nanocomposites and Their Nanocomposite Foams

**Figure 9:**
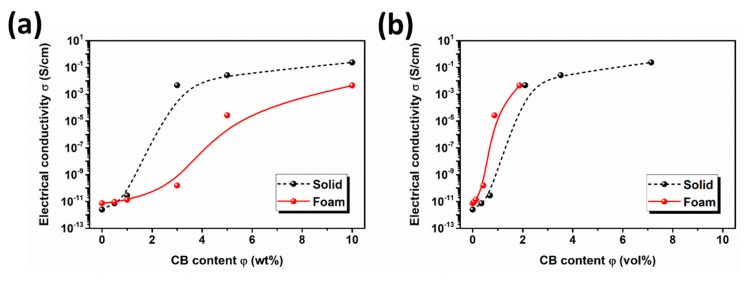
Electrical conductivity of PBS/CB nanocomposites (**a**) and their nanocomposite foams (**b**).

Table 2 lists the density values and CB contents of the solid and foamed samples. Figure 9 shows the electrical properties of the PBS/CB nanocomposites and their nanocomposite foams. As for the PBS/CB nanocomposites (Figure 9a), at a low CB content (0.5 and 1 wt %), the electrical conductivity was improved only slightly. When the CB content was increased to 3 wt %, the electrical conductivity increased to 4.72 × 10^−3^ S/cm, which is 8 orders of magnitude higher than that of neat PBS foams (2.48 × 10^−12^ S/cm). When the CB content was further increased, the electrical conductivity continued to increase by 1 or 2 orders of magnitude before showing a ceiling effect, which indicates that there exists a percolation threshold for PBS/CB nanocomposites with a CB concentration between 1 and 3 wt %. This result also confirms the results of the abovementioned rheological and thermal properties experiment. As for the PBS/CB nanocomposite foams, the electrical conductivity did not show an obvious improvement even when the CB content was 3 wt %. At a CB loading of 5 wt %, the electrical conductivity reached 2.61 × 10^−5^ S/cm, which is 5 orders of magnitude higher than that of PBS3 foams (1.56 × 10^−10^ S/cm). In order to make a comparison between the solid and foamed samples, CB content versus electrical conductivity is shown in Figure 9b in the unit of volume percentage instead of mass percentage. The characteristics of electrical conductivity against CB content in the solid samples exhibited similar tendency curves to those in the foamed samples. However, a lower percolation threshold value (between 0.42 vol % and 0.87 vol %) can be observed for the foamed samples, while the percolation threshold of the non-foamed samples appeared to lie between 0.69 vol % and 2.10 vol %, which provides them with good conductivity performance with a low CB content. Overall, there are two effects of the foaming process on the electrical conductivity percolation threshold. The first effect is that the nanofillers accumulate in the expanded volume of the cells, and the enriching of the nanofillers causes the nanocomposite to have better electrical conductivity. The other effect is that the expansion of the cells’ volume tends to increase the distance between adjacent nanofiller particles. Thus, the percolation threshold of the foamed samples was determined by the degree of volume expansion. In other researchers’ studies on foam, the foaming process was found to reduce the percolation threshold when the volume was expanded by approximately 4–5 times [3,26,27,28]. In this case, the volume expansion of the foam at around 0.13 vol % was ~5.3 times. We believe that the enrichment of CB during the foaming process will reduce the percolation threshold and help to successfully construct a conductive network with the low content of CB nanofiller.

## 4. Conclusions

In this work, the morphology and properties of PBS/CB nanocomposites and their nanocomposite foams were investigated. The CB nanofillers were found to be well dispersed in the nanocomposites even when the CB content was high. With the increase in CB content, the properties of the PBS/CB nanocomposites were found to be significantly enhanced with respect to the crystallization temperature, crystallinity, thermal stability, storage modulus, and complex viscosity. However, the thermal stability and toughness appeared to decrease at a high CB content due to the agglomeration of nanoparticles. In the solid-state supercritical carbon dioxide foaming process, the CB nanofiller showed excellent nucleation efficiency, which caused the cells to be smaller in size and a higher cell density at a high CB content. The resulting PBS/CB nanocomposite foams also exhibited good compressive strength as compared to the neat PBS foams. Furthermore, both the PBS/CB nanocomposites and the nanocomposite foams exhibited good electrical conductivity, and we found that the introduction of a microcellular structure not only reduced the density of the materials but also improved the electrical conductivity. Overall, the obtained PBS/CB nanocomposite foams could be used as lightweight, high-strength, and electrically conductive materials in a wide range of electronics applications.

## Figures and Tables

**Table 1:** Summary of the TGA results for PBS/CB nanocomposites.

Sample	*T*_5%_ (°C)	*T*_max_ (°C)
PBS	321.5	388.1
PBS0.5	323.0	386.1
PBS1	324.0	384.4
PBS3	325.3	387.8
PBS5	329.0	390.3
PBS10	326.4	387.2

**Table 2:** Density values of PBS/CB nanocomposites and nanocomposite foams with different CB contents.

CB Content in Bulk (wt %)	CB Content in Bulk (vol %)	Density of Bulk (g·cm^−3^)	Density of Foam (g·cm^−3^)	CB Content in Foam (vol %)
0	0	1.26	0.107	0
0.5	0.35	1.26	0.205	0.06
1	0.69	1.27	0.237	0.13
3	2.10	1.28	0.258	0.42
5	3.52	1.29	0.319	0.87
10	7.14	1.32	0.344	1.87

## Supplementary Materials

The following are available online at https://www.mdpi.com/2073-4360/11/11/1852/s1, Figure S1: CB particles aggregation size distribution in PBS/CB nanocomposites: (a) PBS0.5, (b) PBS1, (c) PBS3, (d) PBS5, (e) PBS10., Table S1: Summary of the DSC results for PBS/CB nanocomposites.
