# Supplementary material for: Fabrication of Poly(butylene succinate)/Carbon Black Nanocomposite Foams with Good Electrical Conductivity and High Strength by a Supercritical CO2 Foaming Process
Source: Polymers (Basel). 2019 Nov 10;11(11):1852. doi: 10.3390/polym11111852 (PMC6918140; doi:10.3390/polym11111852)
Supplement: Supplementary file 1 [file polymers-11-01852-s001.pdf]

Supplementary Materials

Fabrication of Poly(butylene succinate)/Carbon Black Nanocomposite Foams with Good Electrical Conductivity and High Strength by a Supercritical CO_2 Foaming Process

Zhou Chen[1], Junfeng Hu[1, 3], Jiajun Ju[4] and Tairong Kuang[2,3*]

[1]School of Mechanical and Power Engineering, Nanjing Tech University, Nanjing 211800, China

[2]College of Material Science and Engineering, Zhejiang University of Technology, Hangzhou 310014, China

[3]Suzhou Yi He Yong Li New Energy Co., Ltd, Suzhou 215400, China

[4]The Key Laboratory of Polymer Processing Engineering of Ministry of Education, South China University of Technology, Guangzhou 510640, China

*Correspondence: ktrmonarch0914@gmail.com; kuangtr@zjut.edu.cn

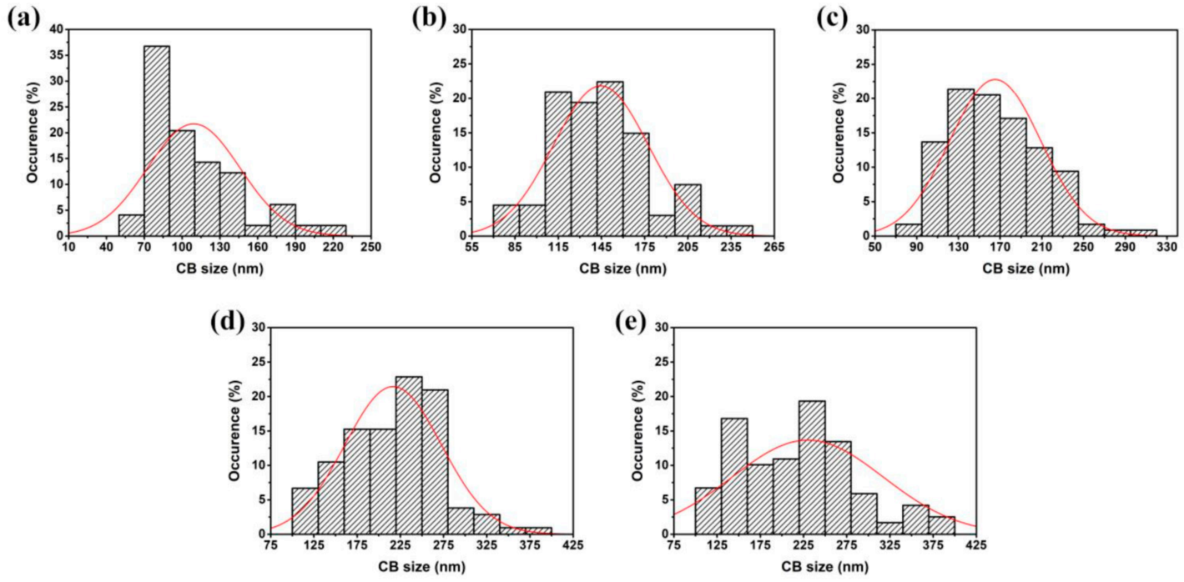

Figure S1. CB particles aggregation size distribution in PBS/CB nanocomposites: (a) PBS0.5, (b) PBS1, (c) PBS3, (d) PBS5, (e) PBS10.

Table S1. Summary of the DSC results for PBS/CB nanocomposites.

Sample	T_{m1} (°C)	T_{m2} (°C)	T_c (°C)	ΔH_m (J/g)	χ_c (%)
PBS	79.6	93.0	52.1	38.6	19.3
PBS0.5	80.1	93.0	54.3	38.8	19.5
PBS1	80.5	93.0	55.2	39.2	19.8
PBS3	80.9	93.0	56.8	39.4	20.3
PBS5	82.1	93.0	57.9	38.8	20.4
PBS10	83.4	93.0	59.2	37.1	20.6
